# Detection and genotyping of *Toxoplasma gondii* DNA in the blood and milk of naturally infected donkeys (*Equus asinus*)

**DOI:** 10.1186/1756-3305-7-165

**Published:** 2014-04-03

**Authors:** Francesca Mancianti, Simona Nardoni, Roberto Papini, Linda Mugnaini, Mina Martini, Iolanda Altomonte, Federica Salari, Carlo D’Ascenzi, Jitender P Dubey

**Affiliations:** 1 Dipartimento di Scienze Veterinarie, Viale delle Piagge, 2 56100 Pisa, Italy

**Keywords:** *Toxoplasma gondii*, Donkey, Seroprevalence, Milk, Genotype, Italy

## Abstract

**Background:**

*Toxoplasma gondii* is a worldwide zoonotic protozoan. Consumption of raw milk from infected animals is considered a risk factor for acquiring toxoplasmosis in humans. Recently, donkey milk has been indicated for therapeutic and nutritional purposes and *T. gondii* infection is common in donkeys. The purpose of the present paper was to detect the presence of parasite DNA in milk of *T. gondii* positive donkeys.

**Findings:**

Antibodies to *T. gondii* were found in 11 out of 44 healthy lactating donkeys by IFAT. *T. gondii* DNA was detected by PCR in blood of 6 and milk of 3 seropositive jennies. Results of limited RFLP-PCR genotyping indicated the presence of *T. gondii* genotype II or III, commonly found in Europe.

**Conclusions:**

The occurrence of *T. gondii* DNA in milk suggests that the consumption of raw milk from seropositive donkeys could be a potential source of human infection.

## Findings

Infections with *Toxoplasma gondii* are widely prevalent in humans and animals, especially food animals throughout the world [1]. Infections are usually asymptomatic in immunocompetent individuals but vertical transmission in humans can lead to the risk of stillbirth, fetal death in utero, or severe central nervous system involvement in newborns, such as cerebral calcifications and hydrocephalus [2]. In immunocompromised individuals toxoplasmosis may cause encephalitis, pneumonitis and life-threatening disease [3]. Drinking raw goat milk has been identified as one of the risk factors for acquiring postnatal toxoplasmosis in humans and pigs [1].

During the last five decades, Italian autochthonous donkeys (*Equus asinus*) suffered from a severe reduction in population size, which led to the extinction of six breeds [4]. At present, nine breeds remain in this country, all classified by FAO as critically endangered or endangered. These include the Asino Amiatina, Asino Argentato di Sologno, Asino Asinara, Asino di Martina Franca, Asino di Pantelleria, Asino Ragusana, Asino Romagnolo, Asino Sardo, and Asino Grigio Siciliano [4]. As a consequence, Italian public institutions and private breeders associations made strong efforts to preserve the still existing autochthonous breeds [5]. Following this strategy, efforts are being made to promote the use of all the remaining Italian donkey breeds in recreational activities (agritourism, trekking) and onotherapy (especially with children) as well as in meat (salami, stew) and milk (cosmetics industry, human nutrition) production [4-6]. The composition of donkey milk is the closest to woman’s milk (relatively poor in protein and fat but rich in lactose) and can be used as a possible substitute for babies, children and adults with Ig E-mediated cow milk allergy [7]. Donkey milk is also recommended as an aid in the prevention of atherosclerosis [8] and tumor therapy [9].

In Italy, farmers are required to follow good hygienic practices but there are no specific rules for donkey breeding and milk production [6]. According to the existing European legislation (reg. EC 853/ 2004 and 1662/2006), raw milk from any species can be sold immediately after milking and directly by the producer to the consumer, or to a local milk seller which in turn is the supplier to final consumers, without any thermal treatment except refrigeration between 0 and 4°C [10]. Given that, recently donkey milk has been increasingly rediscovered as an appreciated source of nourishing food for humans [6], knowledge on safety, quality and hygiene of this milk may become of crucial importance to some categories of consumers (cases of low immune system defenses, elderly, convalescence, infants with cow milk allergy when breast-feeding is not possible). The quality of raw donkey milk has been evaluated microbiologically [11]. Recently, viable *T. gondii* has been detected in raw milk from cows, sheep, goats, buffaloes, and camels [12]. The aim of the present study was to detect *T. gondii* in donkey milk.

In the present study, blood and milk specimens from 44 adult lactating jennies (Asino Amiatina breed, 6 to 14 years old) were obtained during winter 2013. The animals were semi-intensively farmed in paddocks and were healthy, as confirmed by general physical examination. The Asino Amiatina breed was chosen arbitrarily since Tuscany is the top region in terms of population of these donkeys in Italy [4]. Antibodies to *T. gondii* were assayed by immunofluorescent antibody test (IFAT), using commercially available antigen coated 12 well slides (VMRD Inc., Pullman, Washington, USA) and anti-horse-IgG FITC antibody produced in rabbit (Sigma-Aldrich; PBS dilution 1:32). All serum samples were screened at a dilution of 1:20, and positive sera were end-titrated using 2-fold dilutions. After results of serological tests were known, blood samples from seropositive jennies were processed for DNA extraction and subsequent amplification by nested-PCR (n-PCR) as previously described [13], while samples from seronegative jennies were discarded. Similarly, when results on blood samples were known, milk samples (50 ml) from n-PCR positive jennies were processed as above, while samples from negative jennies were discarded. Milk sampling was performed under sterile condition; teats were cleaned and wiped, and 3 squirts of milk were discarded prior to collection in sterile single use plastic vials. Milk contains minor quantities of nucleated cells in comparison to whole blood, so prior to DNA extraction, concentration was carried out by centrifugation at 2200 g for 5 minutes [14]. To avoid interference by casein, 1 ml of pellet was treated with 200 μl TE [1 mM EDTA, 10 mM Tris–HCl (pH = 7.6)] and 300 μl 0.5 M EDTA (pH = 8), then it was resuspended and centrifuged at 3000 g for 10′ [15]. Somatic cells were diluted in 200 μl of PBS and DNA was extracted from both blood and milk somatic cells using the QIAamp® DNA minikit (Qiagen, Milan, Italy) in accordance with the manufacturer’s instructions. The thermic cycle step at 94°C for 5′ we used also denatures the lactoperoxidase present in milk; lactoperoxidase can act against the Taq DNA Polymerase in PCR based-methods. Genotypic characterization of *T. gondii* DNA was performed by PCR amplification of 12 genetic markers (SAG1, 3-SAG2, 5-SAG2, SAG2 new, SAG3, BTUB, GRA6, C22-8, C29-2, L358, PK1, and Apico) as reported [16].

Antibodies to *T. gondii* were found in 11 out of 44 donkeys with antibody titers of 1/160 (n = 2), 1/80 (n = 1), 1/40 (n = 3) and 1/20 (n = 5). *T. gondii* DNA was recovered from blood of 6 and milk of 3 seropositive donkeys (aged 8, 11 and 14 years, respectively). Results of IFAT and n-PCR are summarized in Table 1. Results of genotyping are shown in Table 2. Although we did not get amplification with all markers, available data indicated the presence of genotype III (n = 5) or II (n = 1). To the best of our knowledge, this is the first report of *T. gondii* DNA in blood and milk samples from donkeys and its genotyping in this host species.

**Table 1:** ***Toxoplasma gondii*-antibody titers and results of molecular analysis as determined by immunofluorescence antibody test (IFAT) and nested-PCR (n-PCR) performed on serum, blood and milk samples of lactating jennies, respectively**

**Sample ID**	**IFAT titers**	**Blood n-PCR**	**Milk n-PCR**
1	1/40	Positive	Positive
2	1/160	Positive	Positive
3	1/40	Positive	Positive
4	1/160	Positive	Negative
5	1/80	Negative	Not performed
6	1/20	Negative	Not performed
7	1/20	Negative	Not performed
8	1/20	Positive	Negative
9	1/20	Negative	Not performed
10	1/40	Positive	Negative
11	1/20	Negative	Not performed

**Table 2:** ***Toxoplasma gondii *** **genotypes identified in blood and milk of PCR positive jennies**

**Sample identification nos.**	**Genetic markers**											
	**SAG1**	**3′SAG2**	**5′SAG2**	**SAG2 new**	**SAG3**	**ΒTUB**	**C22-8**	**C29-2**	**GRA6**	**L358**	**PK1**	**Apico**
1	ND	III	III	III	III	ND	III	III	ND	III	ND	III
2	ND	III	III	III	III	III	III	III	ND	III	ND	ND
3	II	II	II	ND	II	ND	II	II	ND	ND	II	ND
4	III	III	ND	III	III	III	ND	III	III	ND	ND	III
8	ND	III	ND	III	III	ND	ND	III	ND	ND	ND	ND
10	ND	III	III	III	ND	ND	ND	III	III	ND	III	ND

ND = No data.

The seroprevalence of *T. gondii* infection in donkeys is frequently high, including seropositivity rates of 45% [17] and 65.6% [18] in Egypt, 43.2% in Brazil [19], 34% in Spain [20], 20.3% [21] and 23.6% [22] in China, from 5 to 8% in Italy [23], and 6.4% in the United States of America [24]. Additionally, milk was found to be positive for *T. gondii* antibodies in 46.3% of pregnant jennies [17]. In our study, *T. gondii* antibodies were found in 25% of serum samples from lactating jennies with *T. gondii* DNA in 13.6% and 6.8% of blood and milk samples from them, respectively. Therefore, a high proportion (27.3%) of lactating seropositive jennies were carrying *T. gondii* DNA in milk. *T. gondii* infection in lactating jennies may result in intermittent excretion of *T. gondii* in milk, as suggested in 3 cases (4, 8 and 10) where the results of n-PCR in blood samples were positive but their results in milk were negative. The stage of *T. gondii* excreted in milk of any animal is unknown, but presumed to be the tachyzoite. We did not make any attempt to determine whether *T. gondii* DNA in lactating jennies was from viable organisms. In a recent report from Iran, 5.7% of raw milk samples from bovine, ovine, caprine, buffalo and camel herds were reported to have viable *T. gondii* as determined by bioassays [12]. Consumption of raw milk has been epidemiologically linked to clinical toxoplasmosis in humans, sometimes with serious consequences [1]. This report adds some hints to this parasite’s knowledge, together with results obtained with other approaches [25], even if further studies are needed to determine viability and the stage of *T. gondii* excreted in milk.

Results of the present study and those of others [12] suggest the possibility of transmission of toxoplasmosis to humans following consumption of raw milk from animals other than the goat.

## Competing interests

The authors declare that they have no competing interests.

## Authors’ contributions

FM designed and performed experiments. RP and CDA participated in designing the study. FM and SN analyzed data and wrote the manuscript. LM carried out PCR detection and genotyping, MM, IA and FS performed fieldwork, SN carried out serological analysis, RP revised results, and JPD critically revised the manuscript. All authors read and approved the final manuscript.

## References

[B1] Jones JL Dubey JP Foodborne toxoplasmosis Clin Infect Dis 2012 55 845 851 10.1093/cid/cis508 22618566

[B2] Havelaar AH Kemmeren JM Kortbeek LM Disease burden of congenital toxoplasmosis Clin Infect Dis 2007 44 1467 1474 10.1086/517511 17479945

[B3] Ajzenberg D Yera H Marty P Paris L Dalle F Menotti J Aubert D Franck J Bessières MH Quinio D Pelloux H Delhaes L Desbois N Thulliez P Robert-Gangneux F Kauffmann-Lacroix C Pujol S Rabodonirina M Bougnoux ME Cuisenier B Duhamel C Duong TH Filisetti D Flori P Gay-Andrieu F Pratlong F Nevez G Totet A Carme B Bonnabau H Genotype of 88 *Toxoplasma gondii* isolates associated with toxoplasmosis in immunocompromised patients and correlation with clinical findings J Infect Dis 2009 199 1155 1167 10.1086/597477 19265484

[B4] Kugler W Grunenfelder H-P Broxham E Donkey breeds in Europe: inventory, description, need for action, conservation. Report 2007/2008 Monitoring Institute for Rare Breeds and Seeds in Europe/SAVE Foundation: St Gallen, Switzerland

[B5] Colli L Perrotta G Negrini R Bomba L Bigi D Zambonelli P Verini Supplizi A Liotta L Ajmone-Marsan P Detecting population structure and recent demographic history in endangered livestock breeds: the case of the Italian autochthonous donkeys Anim Genet 2013 44 69 78 10.1111/j.1365-2052.2012.02356.x 22506921

[B6] Milonis E Polidori P Latte di asina: produzione, caratteristiche, gestione delle aziende Fondazione Iniziative Zooprofilattiche e Zootecniche, Brescia, 2011

[B7] Polidori P Vincenzetti S Use of donkey milk in children with cow’s milk protein allergy Foods 2013 2 151 159 10.3390/foods2020151 PMC5302262 28239105

[B8] Tafaro A Magrone T Jirillo F Immunological properties of donkey’s milk: its potential use in the prevention of atherosclerosis Curr Pharm Des 2007 13 3711 3717 10.2174/138161207783018590 18220810

[B9] Mao X Gu J Sun Y Xu S Zhang X Yang H Ren F Anti-proliferative and anti-tumor effect of active components in donkey milk on A549 human lung cells Int Dairy J 2009 19 703 708 10.1016/j.idairyj.2009.05.007

[B10] Salimei E Fantuz F La produzione di latte di asina in una innovativa filiera latte per consumatori di fascia sensibile Riv Sc Alim – J Food Sci Nutr 2010 39 25 39

[B11] Sarno E Santoro AML Di Palo R Costanzo N Microbiological quality of raw donkey milk from Campania Region It J Anim Sc 2012 11 266 269

[B12] Dehkordi FS Borujeni MR Rahimi E Abdizadeh R Detection of *Toxoplasma gondii* in raw caprine, ovine, buffalo, bovine, and camel milk using cell cultivation, cat bioassay, capture ELISA, and PCR methods in Iran Foodborne Pathog Dis 2013 10 120 125 10.1089/fpd.2012.1311 23441913

[B13] Jones CD Okhravi N Adamson P Tasker S Lightman S Comparison of PCR detection methods for B1, P30, and 18S rDNA genes of *T. gondii* in aqueous humor Invest Ophthalmol Vis Sci 2000 41 634 644 10711675

[B14] Murphy MA Shariflou MR Moran C High quality genomic DNA extraction from large milk samples J Dairy Res 2002 69 645 649 12463700 10.1017/s0022029902005848

[B15] Psifidi A Dovas ID Banos G A comparison of six methods for genomic DNA extraction suitable for PCR-based genotyping applications using ovine milk samples Mol Cell Probes 2010 24 93 98 10.1016/j.mcp.2009.11.001 19900535

[B16] Su C Shwab EK Zhou P Zhu XQ Dubey JP Moving towards an integrated approach to molecular detection and identification of *Toxoplasma gondii* Parasitology 2010 137 1 11 10.1017/S0031182009991065 19765337

[B17] Haridy FM Shoukry NM Hassan AA Morsy TA ELISA-seroprevalence of *Toxoplasma gondii* in draught horses in Greater Cairo, Egypt J Egypt Soc Parasitol 2009 39 821 826 20120748

[B18] El-Ghaysh A Seroprevalence of *Toxoplasma gondii* in Egyptian donkeys using ELISA Vet Parasitol 1998 80 71 73 10.1016/S0304-4017(98)00177-0 9877073

[B19] de Oliveira E de Albuquerque PP de Souza Neto OL Faria EB Júnior JW Mota RA Occurrence of antibodies to *Toxoplasma gondii* in mules and donkeys in the northeast of Brazil J Parasitol 2013 99 343 345 10.1645/GE-3210.1 22924911

[B20] García-Bocanegra I Cabezón O Arenas-Montes A Carbonero A Dubey JP Perea A Almería S Seroprevalence of *Toxoplasma gondii* in equids from Southern Spain Parasitol Int 2012 61 421 424 10.1016/j.parint.2012.02.003 22366344

[B21] Miao Q Wang X She LN Fan YT Yuan FZ Yang JF Zhu XQ Zou FC Seroprevalence of *Toxoplasma gondii* in horses and donkeys in Yunnan Province, Southwestern China Parasit Vectors 2013 6 168 10.1186/1756-3305-6-168 23742078 PMC3679964

[B22] Yang N Mu MY Yuan GM Zhang GX Li HK He JB Seroprevalence of *Toxoplasma gondii* in slaughtered horses and donkeys in Liaoning province, northeastern China Parasit Vectors 2013 6 140 10.1186/1756-3305-6-140 23680297 PMC3659062

[B23] Machacova T Bartova E DI Loria A Sedlak K Mariani U Fusco G Fulgione D Veneziano V Dubey JP Seroprevalence of *Toxoplasma gondii* in donkeys (*Equus asinus*) in Italy J Vet Med Sci 2013 in press 10.1292/jvms.13-0352 PMC3982808 24107428

[B24] Dubey JP Ness SL Kwok OC Choudhary S Mittel LD Divers TJ Seropositivity of *Toxoplasma gondii* in domestic donkeys (*Equus asinus*) and isolation of *T. gondii* from farm cats Vet Parasitol 2014 199 18 23 10.1016/j.vetpar.2013.09.027 24140163

[B25] McGovern KE Wilson EH Dark side illuminated: imaging of *Toxoplasma gondii* through the decades Parasit Vectors 2013 6 334 10.1186/1756-3305-6-334 24267350 PMC4176259

